# Implantation

**Published:** 1887-07

**Authors:** 


### IMPLANTATION.

So far as we can learn, the operations made by Dr. W. J. Younger, of San Francisco, at the east during his visit here last autumn, have not proved invariably successful. The case in Buffalo was not perhaps a fair instance by which to judge of the practice as a whole. The patient was young and healthy, but the implanted tooth had been extracted for some years, and had rested in an unclean place during all the interim. For a time it remained firm and appeared to be doing well. Then it was noticeably less stable, and this mobility increased until it finally fell out, despite the apparatus by which it was attempted to hold it in position. There was never any soreness or inflammation in or about the artificial socket—indeed, we should have felt more hopeful for the final result had a moderate degree of inflammation succeeded the implantation, for it would have been an indication of some functional activity in the tissues.

Upon its removal the tooth gave no evidence of being possessed of a pericemental membrane, nor of any degree of vitality in the cementum. There had, however, been a resorption of the surface of one side of the lower third of the root, which had left it in a honey-combed condition. We were very much disappointed in not having the opportunity to make a microscopical examination of the tooth tissues, but the patient carried it away and lost it.

The tooth implanted for "Archie," an employee of the S. S. White Dental Manufacturing Company, at their depot in Boston, during the meeting of the New England Dental Society in that city last October, also dropped out some time after its implantation. We know nothing of the history of the case, aside from the fact of its failure. It does not necessarily follow that all implantations are failures, but these instances certainly teach that physiological law cannot be violated with entire impunity, and that there are factors which have been overlooked or ignored. Even if every operation made by Dr. Younger at the east should result in failure, the profession will still be under obligations to him for calling attention to the fact that nature will sometimes submit and adapt herself to strange circumstances.

